# In vitro fertilization pregnancy may cause fetal thymic volume involution: A case-control study

**DOI:** 10.18502/ijrm.v21i10.14537

**Published:** 2023-11-24

**Authors:** Nurgul Selin Kaya, Emine Seda Guvendag Guven, Suleyman Guven

**Affiliations:** Departments of Obstetrics and Gynecology, Faculty of Medicine, Karadeniz Technical University, Trabzon, Turkey.

**Keywords:** Fetus, Fertilization in vitro, Prenatal ultrasonography, Thymus.

## Abstract

**Background:**

The effect of modern infertility treatment modalities on fetal thymic volume has not been well known.

**Objective:**

3-dimensional (3D) fetal thymus volumes of 18-24 wk in vitro fertilization (IVF) pregnancies and spontaneous pregnancy cases were compared.

**Materials and Methods:**

135 cases were evaluated in this prospective case-control study. The study was conducted between July 2019 and July 2020 at a university hospital in Trabzon, Turkey. Fetal thymus volume was calculated in the pregnant cases included in the study with the help of the virtual organ computer-assisted analysis system included in the advanced ultrasonography system. The fetal thymus volumes were compared between pregnant women with IVF and spontaneous pregnant women.

**Results:**

The fetal thymus size was significantly lower in the IVF pregnancy group than in spontaneous pregnancy cases (p < 0.001). It was found that the fetal complications, such as non-reassuring fetal health status and requirement for neonatal intensive care, were higher in cases who became pregnant after IVF treatment. It was also found that the rate of any pregnancy complication was significantly higher in IVF pregnancy group (p = 0.02).

**Conclusion:**

In light of these results, it may be concluded that small fetal thymus size may be another fetal complication of IVF pregnancies.

## 1. Introduction

Assisted reproductive techniques (ART) define the clinical and laboratory methodologies employed in infertile couples to eliminate and rectify causes that prevent them from achieving pregnancy. Each year, more than 200,000 babies are born with ART globally, and today, this number is approximately 5 million (1, 2).

An increased complication rate has been observed (such as preeclampsia, intrauterine growth restriction (IUGR), abnormal placental location, preterm birth, and low birth weight) in those who become pregnant with ART (3). Studies comparing babies born from pregnancies obtained with in vitro fertilization (IVF) with the general population show that the rate of congenital anomalies is higher in this group than in the general population (4, 5).

The microinjection technique applied to the oocyte in intracytoplasmic sperm injection cycles and the morphological disorders of the sperm/oocyte affect the development quality of the embryo. Physical damage to the cytoplasm may be possible following a traumatic injection. As a result of these potential risks, it is predicted that zygotes obtained following the intracytoplasmic sperm injection procedure are exposed to nonlethal cell damage and impaired embryonic development (6). Previous studies have shown an increased risk of complications, such as preeclampsia, IUGR, abnormal placental location, and low birth weight in those who become pregnant by using ART (3, 7-10).

In pregnancy, the development process of the fetus's defense system during the intrauterine period focuses on adapting to the existing environment. The fetal thymus assumes the most important role in this immune system. One study showed that the thymus is smaller in various fetal and neonatal diseases (11). In literature, limited studies are found on fetal thymus volume in IVF pregnancies. This study had significant methodological problems, was a retrospective study, and its results were based partly on simple and subjective fetal thymus measurement (11, 12). However, considering that the thymus is a 3-dimensional (3D) structure, 2D evaluation may not provide sufficient information. There is no literature study evaluating the use of 3D virtual organ computer-assisted analysis system (VOCAL) measurement for fetal thymus in IVF pregnancies.

The study aimed to compare the sonographic 3D fetal thymus size in second-trimester spontaneous and IVF pregnancies.

## 2. Materials and Methods

Totally 135 cases (18-24 wk pregnant women without any high-risk pregnancy risk factors) were evaluated in this prospective case-control study. The study was conducted between July 2019 and July 2020 at a university hospital in Trabzon, Turkey.

Participants in the study group were divided into 2 groups (n = 37/each): a) conceived with IVF and b) conceived spontaneously. Since it is forbidden in our country to use donated egg or embryo, their own embryo was used in IVF cycles.

A total of 74 healthy pregnant cases were included according to the inclusion and exclusion criteria of the study. The main criteria for inclusion in the study were being of reproductive age (between 18-35 yr of age) and not having any high-risk pregnancy factors and applying for routine pregnancy follow-up. Exclusion criteria for the study were the presence of any systemic disease (diabetes, hypertension, etc.), additional organ disease (heart, adrenal, thyroid, lung, kidney, blood, etc.) or history of drug use other than routine drugs used during pregnancy, or a history of alcohol use, smoking, or abnormal findings in routine biochemical/hematological test results, or abnormal pregnancy findings (multiple pregnancy, presence of anomaly in the fetus, abnormalities in pregnancy screening tests), or unwillingness to participate in research processes. In this way, 135 cases were evaluated and 61 of them were not included in the study because they did not meet the above criteria. Demographic data of all cases (age, height, weight, systolic/diastolic blood pressures, number of previous pregnancies, etc.) and routine fetal ultrasonographic measurement, gestational age determination data, and fetal 3D fetal thymus volume measurement data were recorded. The pregnancy and birth of all pregnant women were performed in our clinic, and pregnancy follow-up, pregnancy complications, and birth information were recorded. Apgar scores at the 1 ${}^{\text{st}}$ and 5 ${}^{\text{th}}$ min during delivery, newborn weights, cord blood pH, and base excess values were recorded.

### Thymus volume measurement

**Figure 1:**
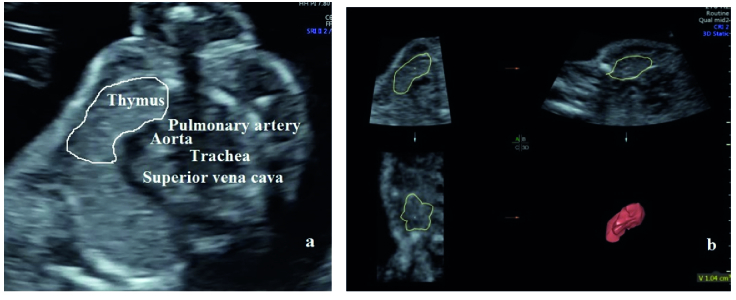
Prenatal sonographic (a) 2- and (b) 3-dimensional fetal thymus view.

Fetal thymus volume was measured with the technique described in detail in our previous study (Figure 1) (13).

Non-reliable fetal status formerly non-reassuring fetal status or fetal distress was defined as persistent or worsening category II or III fetal heart rate pattern.

### Ethical considerations

The study procedure was approved by the Ethical Committee of Karadeniz Technical University, Trabzon, Turkey (Approval no: 2019/205). A written informed consent was obtained from all the participants.

### Statistical analysis

All the data were encoded and entered into SPSS software (version 13.0, Inc., Chicago, Illinois, USA). The statistical analyses were made on the computer using the Chi-square and Student *t* test. Data from both groups were compared with each other. A p-value of less than 0.05 was considered statistically significant. The data were presented as percentages, ratios, and mean ± SD.

## 3. Results

A total of 74 cases (IVF pregnancy [n = 37], and spontaneous pregnancy [n = 37]) were included in the present study. While the average age was 28.62 ± 3.98 in the IVF pregnancy group, it was 27.41 ± 4.03 in the non-IVF group. In table I, IVF pregnancy data are compared with non-IVF pregnancy data. The fetal thymus size was statistically significantly lower in the IVF pregnancy group than in spontaneous pregnancy cases (0.444 ± 0.229 vs. 1.505 ± 0.620 cm^3^, respectively; p < 0.001). The comparison of IVF and spontaneous pregnancies in the study group in terms of pregnancy complications is given in table II. It was found that the fetal complications, such as non-reassuring fetal health status and requirement for neonatal intensive care, were higher at a statistically significant level in cases who became pregnant after IVF treatment compared to the non-IVF pregnancy group. When evaluated in terms of pregnancy complications, pregnancy complications were found to be higher in IVF pregnancies than in non-IVF pregnancies (27.0% and 5.4%, respectively; p = 0.02).

**Table 1:** The comparison of IVF and spontaneous pregnancies regarding demographic and sonographic factors


**Demographic/sonographic factors**	**IVF pregnancy (n = 37)**	**Spontaneous pregnancy (n = 37)**	**P-value**
Age (yr)*	28.62 ± 3.98	27.41 ± 4.03	0.19
Gravida (n)*	2.32 ± 1.70	2.41 ± 1.04	0.80
Parity (n)*	0.92 ± 0.92 ${}^{\text{a}}$	0.95 ± 0.85	0.89
Body mass index (kg/m^2^)*	25.23 ± 5.10	25.09 ± 4.74	0.90
Pregnancy week at measurement time*	20.69 ± 1.84	21.13 ± 1.79	0.30
EFW (g)*	406.24 ± 167.84	465.22 ± 167.03	0.13
BPD (mm)*	20.75 ± 2.11	21.26 ± 2.17	0.30
FL (mm)*	20.63 ± 2.12	21.14 ± 2.02	0.29
HC (mm)*	20.89 ± 2.12	21.27 ± 2.12	0.44
AC (mm)*	21.02 ± 2.20	21.67 ± 2.25	0.21
Birth week (wk)*	38.65 ± 1.37	38.23 ± 1.66	0.51
Birth week (g)*	3133.75 ± 586.03	3327.14 ± 605.99	0.47
Cordon blood gas pH*	7.32 ± 0.06	7.35 ± 0.05	0.21
Cordon blood gas BE (mmol/L)*	-2.07 ± 1.15 ${}^{\text{a}}$	-1.31 ± 1.74 ${}^{\text{b}}$	0.28
1 Min APGAR score*	7.13 ± 1.36	7.00 ± 1.11	0.81
5 Min APGAR score*	8.50 ± 1.07	8.57 ± 0.94	0.87
Fetal gender**	19 (51.4)	27 (73.0)	0.09
C/S rate**	26 (70.3)	28 (75.7)	0.33
*Mean ± SD data were given. Student *t* test was used for comparison. **Data presented as n (%). Chi-square test was used for comparison. The interquartite ranges were ${}^{\text{a}}$ 2 and ${}^{\text{b}}$ 1.7. IVF: In vitro fertilization, EFW: Estimated fetal weight, BPD: Biparietal diameter, FL: Femur length, HC: Head circumference, AC: Abdominal circumference, BE: Base excess, APGAR: Appearance, pulse, grimace, activity, and respiration, C/S rate: Cesarean section rate			

**Table 2:** The comparison of IVF and spontaneous pregnancies in the study group regarding pregnancy complications


**Pregnancy complications**	**IVF pregnancy (n = 37)**	**Spontaneous pregnancy (n = 37)**	**P-value**
**Preeclampsia **	2 (5.4)	1 (2.7)	0.18
**Non-reliable fetal status **	7 (18.9)	1 (2.7)	0.02
**IUGR **	1 (2.7)	0 (0)	0.73
**Neonatal intensive care requirement**	13 (35.1)	4 (10.8)	0.02
Data presented as n (%). Fisher's exact test. IVF: In vitro fertilization, IUGR: Intrauterine growth restriction			

## 4. Discussion

This study showed that fetal thymus size change, which wss an important part of the fetal defense system, was affected by IVF pregnancies. In this prospective case-control study, fetal thymus size was smaller in IVF pregnancies than in spontaneous pregnancies.

The clinical use of fetal thymus measurement is a relatively new prenatal sonographic marker whose importance is not clearly known. Despite the limited number of cases, pregnancy complications between the 2 groups were compared in order to complete this deficiency in the literature, to contribute to the literature in this context, and to reveal the relationship between fetal thymus measurement and increased pregnancy complications in IVF pregnancies.

It was shown in previous studies that were conducted on fetal thymus that fetal thymus hypoplasia is associated with fetal IUGR, chorioamnionitis because of preterm membrane rupture, and chromosomal anomaly (22q 11.2 deletions) (10, 14-17). As an organ, the fetal thymus is sensitive to age and stress-related involution (18, 19). Stress-induced involution is characterized by acute loss of CD 4/8 expression in cortical thymocytes and the peripheral absence of natural T lymphocytes. It is associated with infection, sepsis, trauma, malnutrition, acute respiratory distress syndrome, and physical stress (20, 21). In cases of acute stress due to infection and non-infection, there was a histological reduction in the thymus volume. The thymus is very important for the maturing immune system (22).

Although the effects of impaired thymus development on life-long immunological competence in fetuses that have growth restriction were not elucidated completely, one prospective study shows that immune function in later life is programmed in the early period (23). The result of a study suggested that the thymus is a mediator in the relation between atopic, autoimmune, and infectious diseases in infancy and childhood and fetal malnutrition (24, 25).

Although thymus size was previously investigated in naturally conceived patients having maternal diabetes or rheumatic disease, there are no studies in the literature other than that of Nau and co-workers conducted in 2018 with patients who achieved pregnancy with IVF (12, 26-30).

The study of Nau et al., found that the fetal thymus size was smaller in cases who became pregnant by the IVF method at significant levels when compared to spontaneous pregnancies. Although this study's results support the present study's results, the thymus size was evaluated retrospectively in the abovementioned study, and thymic thoracic ratio was calculated using 2D sonographic measurements (12).

Identifying the fetuses with small thymus will allow the clinician to monitor the patient in this respect, hospitalize if necessary, and anticipate increasing pregnancy complications because of small thymus and IVF pregnancy, and follow-up with such patients closely. The necessary patients can be hospitalized, maternal corticosteroid administration can be applied for lung maturation, and subsequent follow-up may be improved. On the other hand, early intervention for neonatal sepsis and respiratory distress syndrome might reduce perinatal mortality and morbidity by detecting patients with small thymus volumes.

According to a recent study, it has been reported that a 2D ultrasonographic fetal thymus measurement consisting of thymus diameter measurement in a single plane, does not provide effective information about thymus development and evaluation. A 3D-sonographic fetal thymus evaluation can display the fetal thymus more accurately and effectively than standard 2D evaluation providing information about the actual thymic lobular structure and development (28). In this case, our research may more effectively answer the question of whether the commonly used IVF methods cause thymus volume reduction. This makes this research valuable in terms of a new contribution to the literature.

The strong sides of the present study were the design (no additional costs, measurements can be made during routine scanning, and does not require additional hospital visits), each sonogram is measured by the same experienced specialist in the same institution. The thymus is measured in 3D in the most realistic way.

One of the limitations of our study was the small sampling. The number of patients could not be collected at the planned level because of the decreased number of patients who applied to the clinic due to the coronavirus pandemic, which swept the entire world, and the decreased number of patients who underwent IVF treatment.

## 5. Conclusion

The present study showed a relationship between IVF pregnancies and fetal sonographic 3D thymus volume. It was found that the fetal thymus size is significantly lower in cases who become pregnant after IVF treatment compared to spontaneous pregnancies.

It may be concluded that according to the results of this study, fetal thymus 3D volume measurement may be useful in detecting healthy pregnancies and predicting possible neonatal complications. In IVF pregnancies because pregnancy complications are increased, and fetal thymus volume is lower in IVF pregnancy cases at significant levels when compared to spontaneous pregnancies.

##  Conflict of Interest

The authors declare that there is no conflict of interest.
